# Simultaneous Determination of Etomidate and Its Major Metabolite, Etomidate Acid, in Urine Using Dilute and Shoot Liquid Chromatography–Tandem Mass Spectrometry

**DOI:** 10.3390/molecules24244459

**Published:** 2019-12-05

**Authors:** Yu-Kyung Jung, Soo Young You, Seon-Yeong Kim, Jin Young Kim, Ki-Jung Paeng

**Affiliations:** 1 Department of Chemistry, Yonsei University, Wonju 26493, Korea; fts135@yonsei.ac.kr (Y.-K.J.); chaarming@naver.com (S.Y.Y.); kseonyeong@spo.go.kr (S.-Y.K.); 2 Forensic Genetics and Chemistry Division, Supreme Prosecutors’ Office, Seoul 06590, Korea; paxus@spo.go.kr

**Keywords:** etomidate, etomidate acid, metabolite, urine, dilute and shoot, LC-MS/MS

## Abstract

Etomidate (ET) is a commonly used sedative-hypnotic agent such as propofol to induce anesthesia, and it is rapidly metabolized to etomidate acid (ETA) in liver. Herein, a simple method to determine ET and ETA in urine simultaneously was developed using liquid chromatography–tandem mass spectrometry (LC–MS/MS). A simple sample preparation method reduced the total analysis time. For all analytes, the separation was achieved in 6.5 min using reversed-phase chromatography with gradient elution. The best separation and detection of ETA was achieved using a porous graphitic carbon column. The column temperature was maintained at 30 °C to improve the efficiency and sensitivity. The calibration curves were linear over the concentration ranges of 0.4–120.0 ng/mL (ET) and 1.0–300.0 ng/mL (ETA), obtained with a weighting factor of 1/x^2^. The coefficients of determination (r^2^) were greater than 0.9958. The lower limits of quantification were 0.4 ng/mL (ET) and 1.0 ng/mL (ETA), intra-day (n = 6) and inter-day (n = 24) precision values for all compounds were less than 10.2% and 8.4%, respectively, while the intra- and inter-day accuracies were in the −9.9–2.9%, and −7.0–0.6%. The applicability of the method was examined by analyzing the urine samples obtained from ET users.

## 1. Introduction

**Figure 1:**
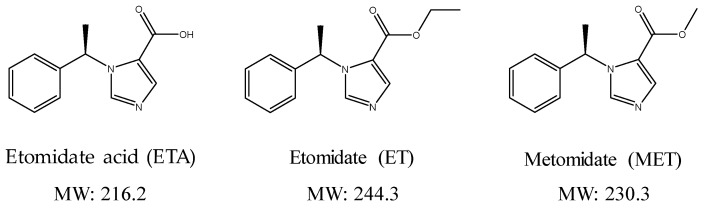
Chemical structures and molecular weights of the analytes (etomidate acid, etomidate) and internal standard (metomidate).

Etomidate (ET) is an intravenous anesthetic used since 1974, which induces sedation and sleep and is used as a whole-body anesthetic agent (Figure 1). Recently, frequent incidents regarding the abuse of ET have been reported because of the increase in the demand for alternative drugs caused by the designation of propofol as a psychosomatic drugs and strict regulations regarding its use. Therefore, it is necessary to develop an analysis method that can effectively monitor ET. As propofol was designated as a drug in 2011, a crackdown in 2012 resulted in the detection of the illegal distribution of propofol by the employees of some pharmaceutical companies to the hospitals and clinics in the area of Gangnam, Seoul, and doctors and entertainers who treated or administered them [1].

ET is a white liquid that is visually similar to propofol and has similar effects. Therefore it has been used as an alternative to propofol, and many smuggling cases have been reported [2].

Several high-performance liquid chromatography (HPLC) and gas chromatography (GC) methods for the analysis of the biomaterials containing ET have been reported, but, each methodology requires either large sample volumes (1–4 mL plasma) or complex sample preparation [3,4,5,6,7,8,9,10]. However, no prior methods have considered the metabolites of ET. The HPLC methods described by Avram [3] and Le Moing [4] afforded acceptable accuracy and precision in the determination of ET concentration, however the methodologies included multiple extraction procedures from the plasma samples. The HPLC methods reported by Ellis [5] and McIntosh [6] included simple and fast extraction procedures, but showed low extraction efficiency (60%), and therefore, a 2 mL of plasma sample was necessary to prevent a high limit of quantitation (LOQ, 50 ng/mL). The ultra-performance liquid chromatography–tandem mass spectrometry UPLC–MS/MS) method proposed by Remane [7] enabled the screening of drugs in the plasma but also showed a high LOQ. The reported GC methods involve the coupling of GC to a variety of detectors that may not be readily available, such as nitrogen–phosphorus detector [8,9] or mass spectrometer [10]. Typically, the sensitivities of these assays are high (1–10 ng/mL), but the plasma sample volumes required are in the range of 1–4 mL, which are generally not suitable for laboratory animal studies.

During the analysis of the medications in blood, an excessive amount of blood is needed, and the collection of the analyte is more difficult than from other specimens, as it requires particular attention in terms of the storage of the specimen. Therefore, urine samples, which are relatively simpler to collect compared to the plasma samples and from which it is easier to extract the analytes during the specimen pretreatment process or analysis samples, are used in this study. ET is rapidly metabolized by esterase in liver, and it metabolites to etomidate acid (ETA) as a hydrophobic drug in phase I and excreted from the kidney [11].

LC–MS/MS is a rapid technique for multi-component screening as it can significantly reduce the sample preparation time [12,13]. The aim of this study is to develop a method for the simultaneous determination of the ET and its metabolite ETA in urine. This is the first report of the simultaneous determination of these analytes. Dilute and shoot LC-MS/MS method, which does not require any pretreatment such as liquid-liquid extraction or solid-phase extraction and specific sample storage conditions is employed. 

In this study, the analytical method is optimized and validated, and its applicability is confirmed by the analysis of the authentic urine samples. This method is simple, rapid and afforded a lower LOQ value compared to the aforementioned analytical methods. 

## 2. Results and Discussion 

Previously reported methods for the determination of ET concentrations in biological fluid samples are complex and time-consuming. The proposed method herein, employing dilute and shoot LC–MS/MS technology, is simple and rapid with sufficient sensitivity and accuracy.

### 2.1. Separation Condition

#### 2.1.1. Column Selection

**Figure 2:**
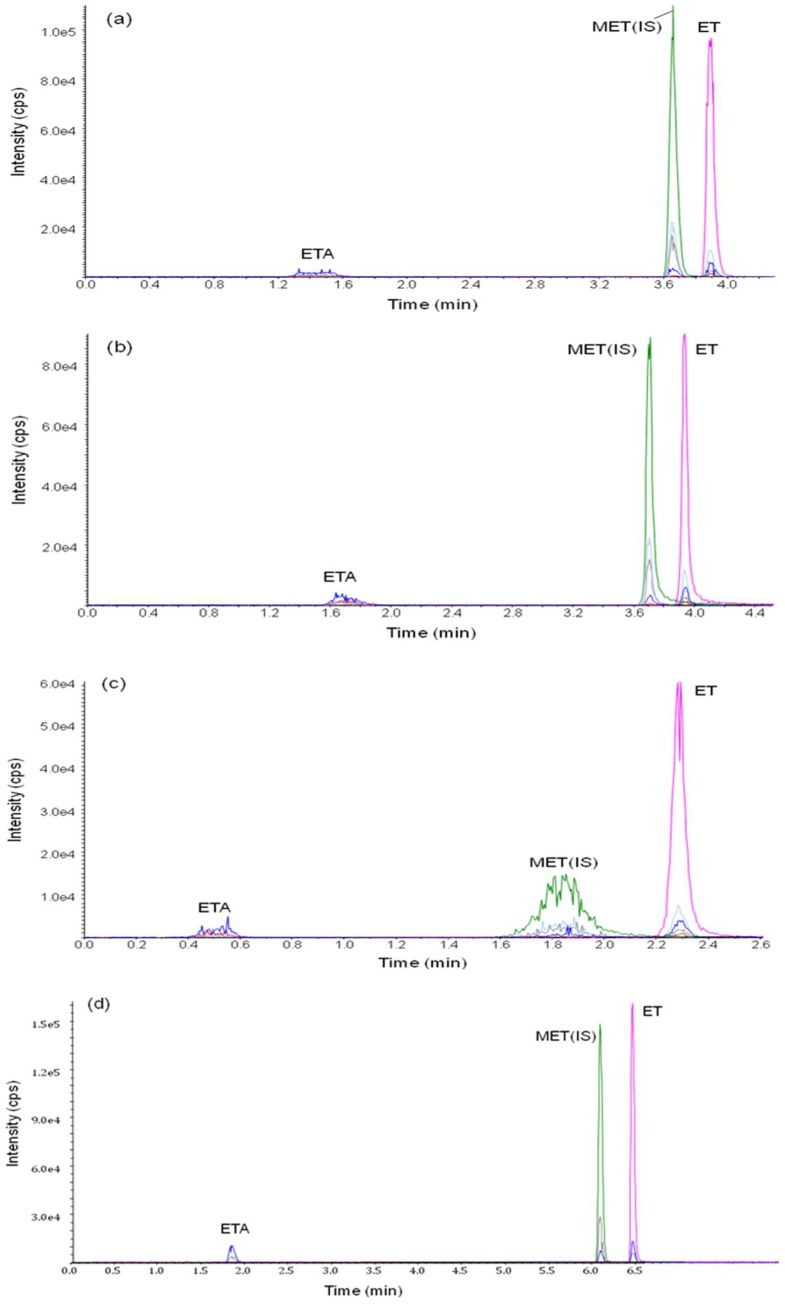
Chromatograms for the separation of etomidate acid and etomidate using four analytical columns: (**a**) C18 column (X-bridge C18, 50 × 2.1 mm I.D., 3.5 μm, Waters Corporation), (**b**) multi-mode C18 column (Scherzo SM-C18, 30 × 2.0 mm I.D., 3.0 μm, Imtakt) (**c**) silica-based polar end-capped C18 column (Hypurity Aquastar , 50 × 2.1 mm I.D., 5.0 μm , Thermo Fisher Scientific), and (**d**) porous graphitic carbon column (Hypercarb, 50 × 2.1 mm I.D., 5.0 μm, Thermo Fisher Scientific). Solvent A (0.05 % formic acid in water) and solvent B (50%:50% acetonitrile:methanol) were used for analysis. The gradient elution included 15% B for 0.4 min, 85% B for 3 min to 5 min, and re-equilibration to 15% B for 7.5.

To separate ET and ETA, four types of columns were tested. Figure 2 shows the representative chromatograms for ET and ETA using these four analytical columns. The parameters such as peak symmetry, resolution, sensitivity, selectivity and pressure stability were considered for the selection of the column. All columns were evaluated using 0.1 μg/mL of the target analytes. 

As shown in Figure 2a–c, the ETA peak is not clearly detected using C18, multi-mode C18 and silica based polar end-capped C18 column. However, an appropriate shape of the ETA peak is observed using the porous graphite carbon (Hypercarb) column (Figure 2d). Additionally, the Hypercarb column is also tolerant toward high pressure due to high flow rate of above 450 μL/min. Therefore, the Hypercarb column was used in this study. 

The column temperature was optimized to increase the peak symmetry. As the column temperature increases, the viscosity of the mobile phase decreases, and the flow rate is high. An increase in the temperature of the column affords a decrease in the retention times (RTs) of the peaks, as well as an improvement in the peak tailing. However, the peak sensitivity decreases with an increase in temperature, particularly for ETA, and the column temperature was set to 30 °C during the analysis.

#### 2.1.2. Mobile-Phase Optimization

Optimal separation conditions were determined for ET and ETA by varying the mobile-phase compositions. Gradient elution with deionized water (solvent A) containing acid and buffer solution and polar organic solvents (solvent B) was used for optimal separation. The gradient conditions are listed in Table 1. The optimum mixing ratio of the elution solvent was determined based on the change in their RT with a variation in the ratio of acetonitrile and methanol. 

**Figure 3:**
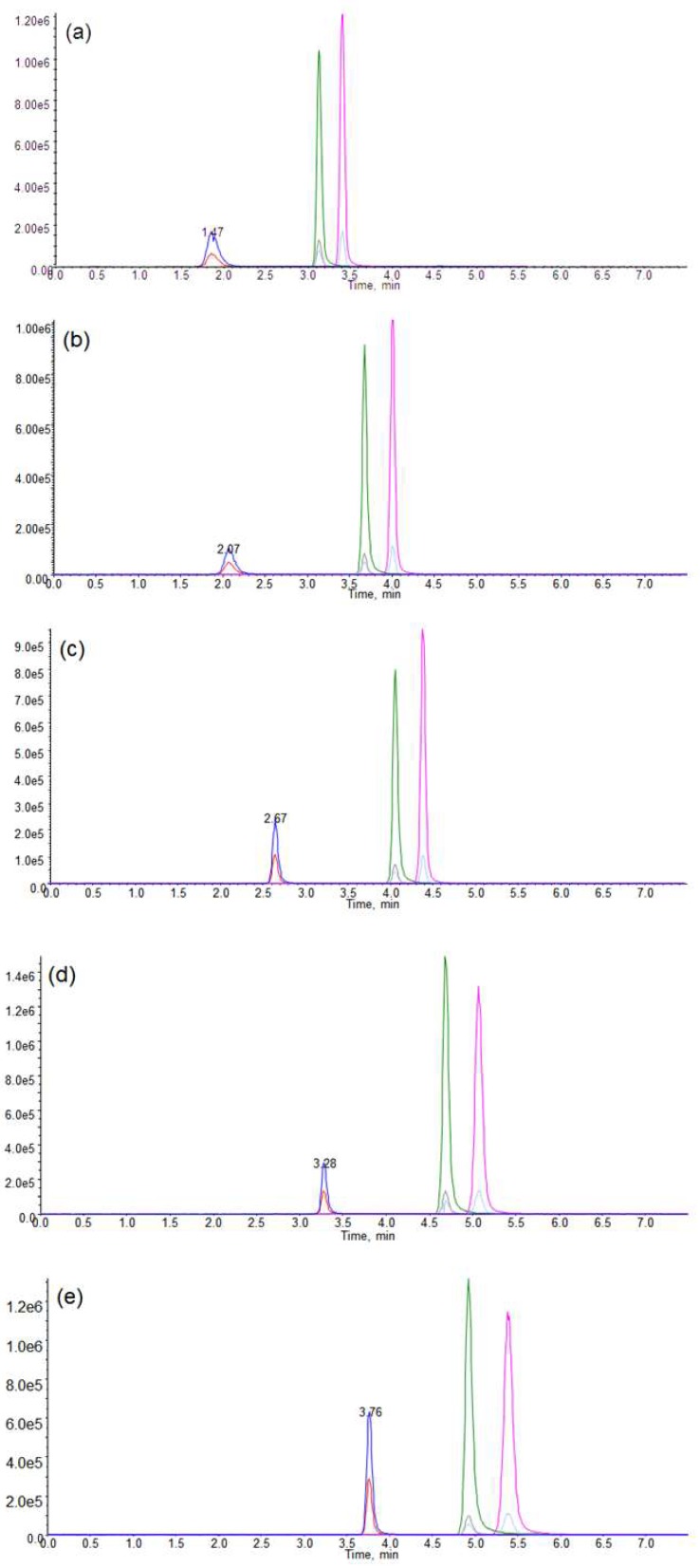
Effect of the changing ratio of acetonitrile and methanol: (**a**) acetonitrile, (**b**) acetonitrile:methanol (v:v, 75:25), (**c**) acetonitrile:methanol (v:v, 50:50), (**d**) acetonitrile:methanol (v:v, 25:75), (**e**) methanol.

Figure 3 shows the representative chromatograms of ET and ETA using five different compositions of the mobile phase. When acetonitrile is used as the mobile phase, high flow rate can be used, and it is easy to control other variables. However, the ETA peak is not observed using acetonitrile as mobile phase B (Figure 3a). Therefore, an increasing ratio of methanol was used. Although the peak intensity and area of ETA increase as the ratio of methanol increases, a high ratio of methanol does not allow the separation of analytes peaks due to tailing. (Figure 3d,e). Thus, acetonitrile and methanol mixture (50:50 v:v) was selected as the elution solvent B (Figure 3c), as the separation of peaks could be achieved without tailing. 

Methanol and acetonitrile are polar solvents, but acetonitrile is considered as a medium polarity solvent and methanol is considered as a polarity solvent. As strong hydrophobicity and low polarity of solvents lead to strong interactions on the graphitic carbon column, the strength of the acetonitrile is higher than methanol as a solvent.

The mobile phase in LC–MS can be modified for efficient analysis by acidification with formic acid or acetic acid with ammonium acetate or ammonium formate as ion pair reagents. The conditions for acids and buffers were optimized by applying these in the positive ion mode.

**Figure 4:**
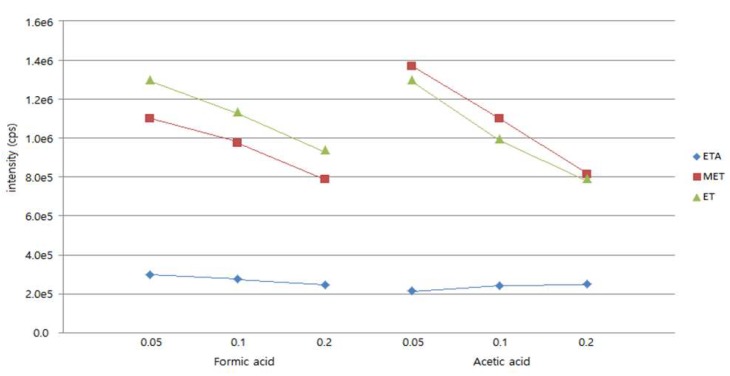
Effect of the concentration of charged additive.

The peak shapes and intensities were compared by adding 0.05%, 0.1%, and 0.2% of each acid to the mobile phase (Figure 4). With an increase in the concentration of formic acid, the peak intensities of all analytes decrease. As the concentration of acetic acid increases, the peak intensities of ET and MTA decrease and that of ETA increases. Based on this result, deionized water with 0.05% formic acid, which afforded optimal sensitivity, was used as solvent A. 

Upon the addition of ammonium acetate or ammonium formate buffer in solvent A, the peaks not well formed and instead show split peaks. Suitable results are not obtained even with the combined addition of acids and buffers. Therefore, buffers were not added in solvent A.

### 2.2. Matrix Effect

**Figure 5:**
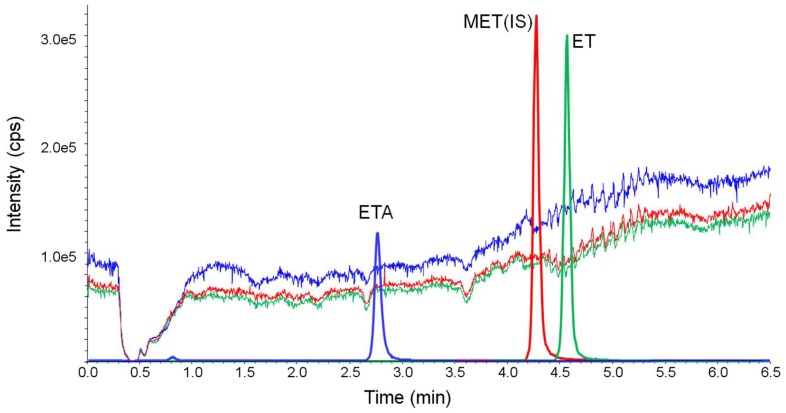
Ion suppression studied by monitoring the multiple reaction monitoring (MRM) chromatograms during post-column infusion of 30 ng/min of etomidate acid (ETA), 2 ng/min of metomidate (MET, internal standard, IS) and ET, and subsequent injection of the diluted urine sample.

The ion suppression was tested through post-column injection to check the matrix effect in the urine samples. Figure 5 shows the multiple reaction monitoring (MRM) chromatograms obtained by injecting blank urine in to the post-column with the standard solution of the mobile phase and analytes flowing through the column at a flow rate of 30 ng/min for ETA and 2 ng/min for ET. As ion suppression occurs for approximately 0.3–1.0 min, the separation conditions prevent the formation of a peak in this RT range. 

### 2.3. Validation

#### 2.3.1. Selectivity, Linearity, Limit of Detection (LOD) and Limit of Quantitation (LOQ)

**Figure 6:**
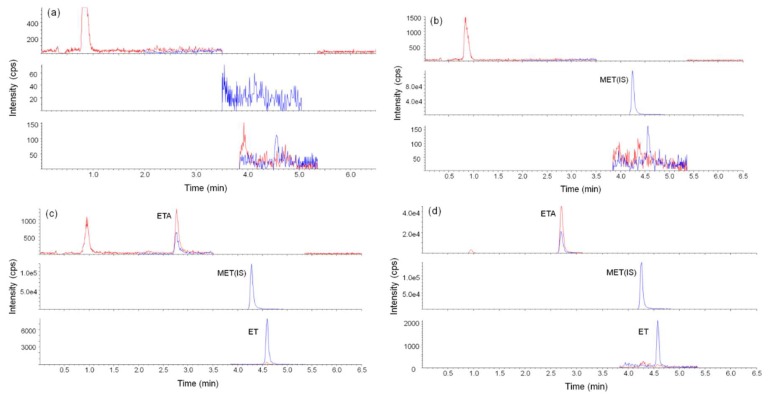
Representative liquid chromatography–tandem mass spectrometry (LC–MS/MS) chromatograms for the (**a**) blank (**b**) blank with metomidate (internal standard (IS)) (**c**) blank with IS, etomidate and etomidate acid, and (**d**) etomidate-positive urine sample.

The selectivity test was performed based on the LC-MS/MS chromatogram. The chromatograms obtained with blank sample (Figure 6a), blank urine sample with only the internal standard (IS, Figure 6b), blank urine sample with the IS and material to be analyzed (Figure 6c) and real sample from ET user (Figure 6d) are shown below. The RTs of the analytes were determined in advance. The RTs of all substances do not overlap with each other and no other interferences are observed low LOQ values. 

The LODs of 0.03 and 0.01 ng/mL and lower LOQs of 0.1 and 04 ng/mL are obtained for ETA and ET, respectively. The range of the calibration curves obtained from LOQ values depends on the sensitivity of the instrument for each analyte. 

A linearity (r^2^) of 0.99 for 0.1–300 ng/mL ETA and 0.4–120 ng/mL ET is observed. The calibration range, slope, y-intercept, r^2^ value, LOD and lower limit of quantification (LLOQ) values for each analyte are listed in Table 2.

#### 2.3.2. Precision and Accuracy

Table 3 includes the precision and accuracy values for each analyte. The accuracy and precision of inter-day (n = 24) and intra-day (n = 18) were obtained by considering the detectable ranges of the samples of each analyte. The inter-day analysis was conducted continuously for four days and intra-day analysis was conducted by repeating the experiment three times in the same day. Intra-day accuracy of −9.9–2.9% and inter-day accuracy of −7.0–0.6%, as well as inter-day precision of 10.2%, and inter-day precision of 8.4% are observed. 

#### 2.3.3. Process Efficiency

The determination of process efficiency (PE) is another method to verify the matrix effect. The percentage value of the PE was calculated by the peak area of the analyte in urine samples divided by the peak area of the analyte in the mobile phase. The peak area of the analyte was measured seven times for the three standard samples and the average values were obtained. Coefficient of variation (CV) is the precision value based on the PE (%) of each standard sample. The identified values of the average PE (%) are 88.5% within 8% CV for ETA and 102.1% within 5% CV for ET. Through PE and post-column injection of the analyte, the matrix effect is determined and the detection part of the analyte is not detected.

The stability tests including bench-top stability, long-term stability, and auto-sampler stability were conducted with low quality control (LQC) and high quality control (HQC) samples of ET and ETA. Bench-top stability is the stability test of the samples under the lab-handling conditions at RT for 6 h. Results include stability below 11.0% for ETA and below −4.7% for ET. Long-term stability tests were conducted at 4 °C for 7 days and 14 days to check the stability of the sample. The stability at 7 days is below −13.3% for ETA and below −7.5% for ET. Additionally at 14 days, the stability is below −20.7% for ETA and −9.7% for ET. The auto-sampler stability was determined with storage at 4 °C for 12 h. The calculated stability is below −12.5% for ETA and −7.0% for ET, respectively. The stability test results are included in Table 4. All results show an accuracy within 15%, except for the low QC of ETA for the 14-days sample. Base on this result, ET and ETA analysis should be conducted within seven days to ensure the stability of the samples.

### 2.4. Method Feasibility 

Two authentic urine samples were analyzed using this method to quantify ET and ETA. The samples from the ET users were provided by Seoul National University, Bundang Hospital.

The concentrations of ET and ETA in urine determined by the developed method are included in Table 5. Based on the data obtained from the analysis of the actual urine samples, the metabolism of ET in urine to ETA is confirmed. 

## 3. Materials and Methods

### 3.1. Reagents

The analytes, ET and ETA, and an IS, metomidate (MET) were purchased from Toronto Research Chemicals (Toronto, ON, Canada). The standard solutions with a concentrations of 100 μg/mL in methanol were prepared and stored at −20 °C. Formic acid, acetic acid, ammonium formate and ammonium acetate that were used as additives in the mobile phase were purchased from Sigma-Aldrich (St. Louis, MO, USA). HPLC grade methanol and acetonitrile were purchased from J.T. Barker (Phillipsburg, NJ, USA) and Burdick & Jackson (Muskegon, MI, USA), respectively. Deionized water was prepared by removing the ions using the Millipore direct-Q water purification system (Billerica, MA, USA) of. The blank urine used in this experiment was urine of a female who did not use etomidate and was obtained voluntarily.

### 3.2. Urine Specimens

Blank urines were obtained with consent by women who had no administer with etomidate. Blank urine was used in the method validation as drug-free urine. Matrix matched calibrators were prepared by adding mixed standard solution to blank urine samples over the concentration range of 1, 2.5, 5, 10, 25, 50, 100, and 300 ng/mL (ETA), 0.4, 1, 2, 4,10, 20, 40 and 120 ng/mL (ET). Quality control (QC) samples were also prepared in the same way, using a separately prepared stock solution at a concentration of 3 (low) and 90 ng/mL (high) for ETA and 1.2 (low), 36 ng/mL (high) for ET.

Forensic urines were provided by Seoul National University, Bundang Hospital in Republic of Korea. Urine samples were stored at 4 °C up to 7 days and then positive urine samples of these were kept separately in a freezer at –20 °C for reanalysis if required.

### 3.3. Analytical Instrument and Conditions

#### 3.3.1. Liquid Chromatography

The 1260 infinity LC system (Agilent Technologies, Palo Alto, CA, USA) was used for the separation of ET and ETA. To select a suitable separation column for the analyte, four types of columns with different fixed surfaces were tested. The columns tested included X bridge C18 (50 × 2.1 mm I.D., 3.5 μm, Waters Corporation, Milford, MA, USA), Scherzo SM-C18 (30 × 2.0 mm I.D., 3.0 μm, Imtakt, Portland, OR, USA), Hypurity Aquastar (50 mm × 2.1 mm I.D., 5.0 μm, Thermo Fisher Scientific, Waltham, MA, USA) and Hypercarb (50.0 mm × 2.1 mm, 5.0 μm, Thermo Fisher Scientific, Waltham, MA, USA). For appropriate separation of the analyte, the column temperature was optimized and maintained for the entire analysis time. 

The mobile phase compositions and the ratios of acid, buffer, and organic solvents were optimized. The composition of the optimized mobile phase included 0.05% formic acid in deionized water for the solvent A and acetonitrile: methanol (50:50, v:v) for organic solvent B. The gradient elution included 15% B for 0.4 min, 85% B for 3 min to 5 min, and re-equilibration to 15%B for 7.5 min (Table 1). The analysis time was 7.5 min and the injection volume was 5 μL. The auto-sampler temperature was maintained at 4 °C to prevent analyte degradation. The Sciex software (version 1.5.1, Sciex, Toronto, ON, Canada) was used for data collection.

#### 3.3.2. Tandem Mass Spectrometry (MS/MS)

The triple quadruple API 3200 (Sciex, Toronto, ON, Canada) with electrospray ionization (ESI) was used in this study. To optimize the detection of [M + H]^+^, the parameters were optimized by the direct infusion of 1 μg/mL of analyte. MRM mode was used to confirm the fragment-ions, and MRM transition, retention time (RT), declustering potential (DP), entrance potential (EP), collision energy (CE), collision cell entrance potential (CEP) and collision cell exit potential (CXP) for the analyte and IS are summarized in Table 6.

**Figure 7:**
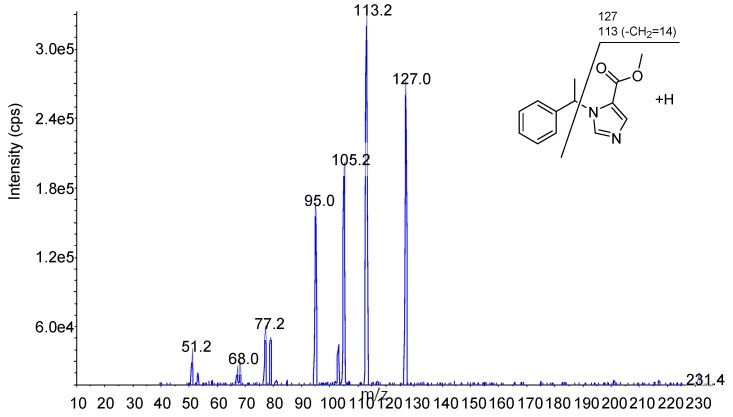

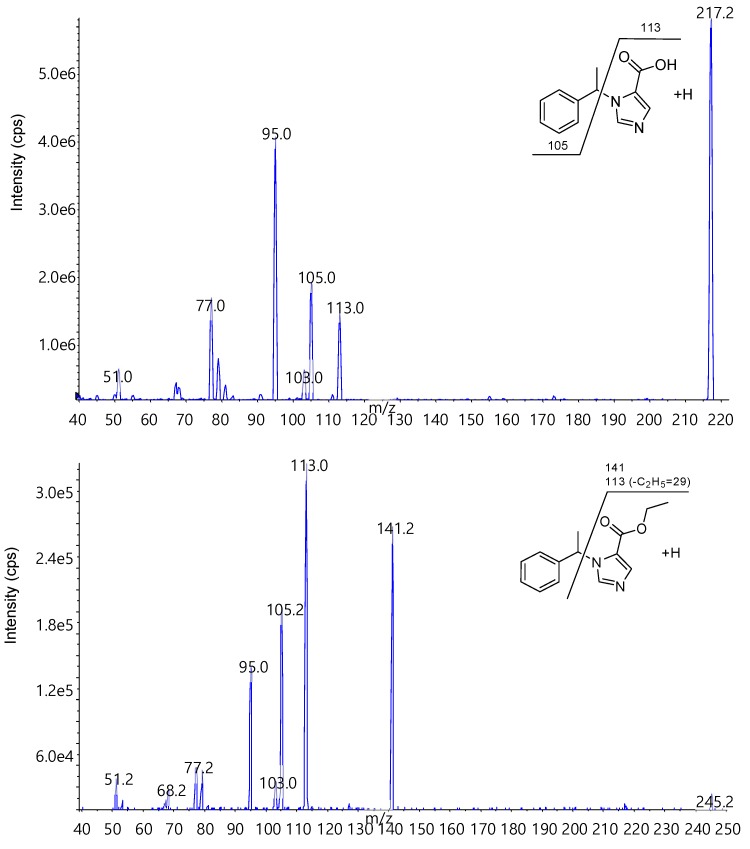
Precursor and product ion of each compound.

The mass spectra of the molecular and fragment ions (Figure 7) for each analyte and IS were obtained under conditions listed in Table 1.

### 3.4. Sample Preparation

Five dilutions of the standard solution were prepared with blank urine in the range of 0.1–300 ng/mL for ETA and 0.4–120 ng/mL for ET. For validation, samples were prepared at concentrations of 1.0, 3.0, 30.0, and 90.0 ng/mL in blank urine for ETA and 0.4, 1.2, 12.0 and 36.0 ng/mL in blank urine for ET, whose concentration corresponded to LLOQ, low quality control (LQC), mid quality control (MQC), and high quality control (HQC), respectively. MET with a concentration of 0.2 ng/mL was used. The urine sample (120 μL) and 0.1 μg/mL IS (30 μL) were placed into a 1.5 mL Eppendorf tube and centrifuged at 50,000× *g* for 3 min. 20 μL of the supernatant was taken, transferred to a LC-MS vial, and 5 μL of this was injected. All subjects provided their informed consent for participation in the study. 

### 3.5. Matrix Effect

The matrix effect was confirmed by post-column injection. The infusion pump injected the target analytes at a constant speed and HPLC injected the blank urine specimen without the target analytes to check the occurrence of ion enhancement or ion suppressions.

### 3.6. Validation

To assess the practical applicability of the analysis method, it was validated following the USFDA Guidance for validation of bioanalytical methods [14]. This analysis method was applied to the actual samples to confirm the practical usefulness.

## 4. Conclusion

In this study, a simple dilute and shoot LC–MS/MS, analysis method was developed for the simultaneous determination of ET and ETA in urine. Notably, the drug and trace amount of its metabolites are analyzed simultaneously in 120 μL of human urine sample, and the analysis can be performed immediately after dilution without further pretreatment. ET and ETA are analyzed within 6.5 min by modifying the mobile phase, column and analysis conditions. 

The method is validated and applicability to the real sample is confirmed by analyzing the urine samples from drug users, which shows the feasibility of monitoring ET intake in humans.

Selectivity is verified based on the data for a blank sample with no added materials. Correlation factors of 0.9963 and 0.9958 for ET and ETS, are observed in the calibrated range of 0.4–120.0 ng/mL for ET and 1.0–300.0 ng/mL for ETA respectively. The LLOQ values are 0.4 ng/mL (ET) and 1.0 ng/mL (ETA), respectively. Intra-day (n = 6) and inter-day (n = 24) precision values for all compounds are less than 10.2% and 8.4%, and the intra- and inter-day accuracies are in the ranges of −9.9–2.9%, and −7.0–0.6%, respectively. The applicability of the method was examined by analyzing the urine samples obtained from ET users.

## Figures and Tables

**Table 1:** Gradient program condition for the separation of analytes.

Total Time (min)	Flow Rate (μL/min)	Mobile Phase	
		Solvent A (%) ^a^	Solvent B (%) ^b^
1	400	85	15
0.4	400	85	15
3	450	15	85
5	450	15	85
5.1	400	85	15
7.5	400	85	15

^a^ Solvent A: 0.05 % formic acid in water; ^b^ Solvent B: acetonitrile: methanol (50:50, v:v).

**Table 2:** Validation parameters for the calibration curves of ETS and ET.

Analyte	Concentration Range (ng/mL)	Slope (mean ± SD)	y-Intercept (mean)	Linearity ^a^ (R^2^)	LOD ^b^ (ng/mL)	LLOQ ^c^ (ng/mL)
Etomidate acid	1.0–300.0	0.0124 ± 0.0008	0.0004	0.9958	0.03	1.0
Etomidate	0.4–120.0	0.1148 ± 0.0098	0.0051	0.9963	0.01	0.4

^a^ Linearity is described by the determination coefficient for the calibration curve. ^b^ Limit of detection (LOD) is based on the concentration corresponding to the signal of three times the standard deviations obtained from the mean of the ten replicates of the blank urine samples. ^c^ Lower limit of quantification (LLOQ) is defined as the lowest concentration in the calibration curve with precision (% CV) less than 20% and accuracy (% bias) within ±20%.

**Table 3:** Intra- and inter-day precision and accuracy.

Analyte	Nominal Concentration (ng/mL)	Intra-Day (n = 18)		Inter-Day (n = 24)	
		Precision ^a^ (% CV)	Accuracy ^b^ (% bias)	Precision (% CV)	Accuracy (% bias)
Etomidate acid	1.0	10.2	2.9	8.4	−6.4
	3.0	4.7	2.4	6.0	−4.1
	30.0	2.5	−0.2	2.3	−1.3
	90.0	1.7	−7.4	2.7	−7.0
Etomidate	0.4	6.3	−9.9	4.1	−5.5
	1.2	3.3	−3.7	3.3	0.6
	12.0	2.0	−4.0	4.0	1.3
	36.0	4.2	−8.6	3.0	−6.1

^a^ Expressed as the relative standard deviation of the peak area ratios of the analytes/internal standard. ^b^ Calculated as [(mean calculated concentration – nominal concentration)/nominal concentration] × 100.

**Table 4:** Stability test results for bench-top stability, long-term stability, and auto-sampler stability.

Compound	Concentration (ng/mL)	Bench-Top Stability (%)	Long-Term Stability (%)		Auto-Sampler Stability (%)
		(Room Temperature for 6 h)	(4 °C for 7 days)	(4 °C for 14 days)	(4 °C for 12 h)
Etomidate acid	3.0	−11.0	−13.3	−20.9	−12.5
	90.0	−10.5	−8.3	−11.8	−9.3
Etomidate	1.2	−0.5	−7.5	−9.7	−7.0
	36.0	−4.7	−5.3	−5.7	−4.5

**Table 5:** Urinary concentrations (ng/mL) of etomidate acid and etomidate for authentic samples.

Sample#	Etomidate Acid Concentration (ng/mL)	Etomidate Concentration (ng/mL)
# 1	23.79	< LLOQ
# 2	31.38	< LLOQ

**Table 6:** Retention times, MRM transitions and compound dependent parameters for LC–MS/MS analysis of the analytes and internal standard (IS).

Analyte	RT ^a^ (min)	MRM Transition		DP ^b^	EP ^c^	CEP ^d^	CE ^e^	CXP ^f^
		Q1	Q3					
Etomidate acid	2.75	217.1	113.1	24	8.5	12	18	6
			105.2	24	8.5	12	37	6
Metomidate (IS)	4.29	231.2	127.1	26	4	12	15	4
			113.2	26	4	12	31	4
Etomidate	4.6	245.2	113.2	26	4.5	12	27	4
			141.2	26	4.5	12	15	6

^a^ RT: retention time. ^b^ DP: declustering potential. ^c^ EP: entrance potential. ^d^ CEP: collision cell entrance potential. ^e^ CE: collision energy. ^f^ CXP: collision cell exit potential.
